# Is there a gender difference in noninvasive coronary imaging? Multislice computed tomography for noninvasive detection of coronary stenoses

**DOI:** 10.1186/1471-2261-8-2

**Published:** 2008-01-29

**Authors:** Marc Dewey, Wolfgang Rutsch, Bernd Hamm

**Affiliations:** 1 Department of Radiology, Charité, Medical School, Humboldt-Universität zu Berlin, Germany; 2 Department of Cardiology, Charité, Medical School, Humboldt-Universität zu Berlin, Germany

## Abstract

**Background:**

Multislice computed tomography (MSCT) coronary angiography is the foremost alternative to invasive coronary angiography.

**Methods:**

We sought to compare the diagnostic accuracy of MSCT in female and male patients with suspected coronary disease. Altogether 50 women and 95 men underwent MSCT with 0.5 mm detector collimation. Coronary artery stenoses of at least 50% on conventional coronary angiography were considered significant.

**Results:**

The coronary vessel diameters of all four main coronary artery branches were significantly larger in men than in women. The diagnostic accuracy of MSCT in identifying patients with coronary artery disease was significantly lower for women (72%) compared with men (89%, *p *< 0.05). Also sensitivity (70% vs. 95%), positive predictive value (64% vs. 93%), and the rate of nondiagnostic examinations (14% vs. 4%, all: *p *< 0.05) were significantly worse for women. The effective radiation dose of MSCT coronary angiography was significantly higher in the examination of women (13.7 ± 1.2 mSv) than of men (11.7 ± 0.9 mSv, *p *< 0.001), mainly as a result of the fact that the radiosensitive female breast (contributing 24.5% of the dose in women) is in the x-ray path.

**Conclusion:**

Noninvasive coronary angiography with MSCT might be less accurate and sensitive for women than men. Also, women are exposed to a significantly higher effective radiation dose than men.

## Background

Since conventional coronary angiography exposes the patients not only to rare (1.7%) but relevant risks like bleeding, stroke, infarction, and dissections but also has a considerable mortality of 0.11% [1] a noninvasive alternative would be an important advance. At present the foremost alternative to conventional coronary angiography is multislice computed tomography (MSCT) [2-7] which has a high spatial (0.5 to 0.75 mm slice thickness) and temporal resolution (140 to 200 ms acquisition window). To reliably exclude the presence of coronary artery stenoses is the primary aim of noninvasive coronary angiography using MSCT [8]. However, MSCT exposes the patient to radiation and requires intravenous injection of a contrast medium. For these reasons and since gender differences play a prominent role in cardiac imaging [9] and therapy [10] it appears worthwhile to examine gender differences of MSCT in detail before routine application of this technology. Thus, we prospectively analyzed the diagnostic accuracy of MSCT in women and men as part of an investigator-initiated study on noninvasive coronary angiography [11].

## Methods

### Study population

A total of 126 patients (Table 1) with suspected coronary artery disease and without contraindications (creatinine above 1.5 mg/dL, allergy to iodinated contrast agents) underwent MSCT and conventional coronary angiography as part of an investigator-initiated trial [11] in which all patients are included in the analysis (intention-to-diagnose design) [12]. Exclusion criteria for the study were age below 40 years (as requested by the Federal Department for Radiation Protection), nonsinus rhythm, previous conventional coronary angiography, pregnancy, breast-feeding, orthopnea, unstable angina, and myocardial infarction. The institutional review board and the Federal Department for Radiation Protection approved the study and all patients gave written informed consent. To increase the amount of female patients available for this comparison (beyond that of the 31 women who were included in the intention-to-diagnose study mentioned above) we included women (19) who underwent CT coronary angiography using 16 detector rows and conventional coronary angiography (both performed before and after CT) at our institution for clinical purposes and included them in the present analysis of gender differences in regards to MSCT coronary angiography (50 women and 95 men).

**Table 1:** Patient characteristics

	Women (n = 50)	Men (n = 95)	p
Age (years)	63.9 ± 8.6	62.6 ± 9.6	0.413^†^
Body mass index*	26.6 ± 4.3	27.3 ± 3.4	0.139^†^
Smokers	11.8%	28.4%	< 0.05
Diabetes mellitus	14.0%	15.8%	0.776^†^
Hyperlipidemia	60.0%	49.5%	0.229^†^
Arterial hypertension	66.0%	73.7%	0.334^†^
Typical angina	40.0%	51.6%	0.186^†^
Atypical angina	30.0%	21.1%	0.233^†^
Results of conventional coronary angiography			<0.001^$^
No disease	40/50 (80%)	36/95 (38%)	
One-vessel disease	6/50 (12%)	13/95 (14%)	
Two-vessel disease	4/50 (8%)	22/95 (23%)	
Three-vessel disease	0	23/95 (24%)	
Four-vessel disease	0	1/95 (1%)	

* Calculated as the weight in kilograms divided by the square of the height in meters.

^† ^not significant.

^$ ^compared using the chi-square test.

### MSCT protocol

Scanning was performed on an MSCT scanner using 16 × 0.5 mm detector collimation (Aquilion 16, Toshiba Medical Systems, Otawara, Japan) as recently described [11] with retrospective ECG gating, multisegment reconstruction [7,13], 0.4 s rotation time, 120 kV, 300 mA, and 0.2 pitch, and an average image reconstruction interval of 146 ms, which was not significantly different between women (149 ± 36 ms) and men (146 ± 37 ms). Nitrate was administered prior to MSCT to increase the coronary artery diameters and to facilitate image assessment [14]. No beta blockers were given since the main purpose of the investigator-initiated study was to compare MSCT and magnetic resonance coronary imaging and beta blockers might have favored CT over magnetic resonance. However, 74 of the 145 patients were on chronic oral beta blocker medication (24 women, 50 men). The manual sure-start feature of the scanner was used to visualize the influx of the intravenous contrast medium (bolus-tracking) and to start image acquisition. The average breathhold time and helical scan length for covering the coronary arteries (from the left atrium to the base of the heart) were both significantly (p < 0.01) shorter for women (28.0 ± 3.0 s and 9.5 ± 1.2 cm) than men (29.6 ± 2.5 s and 10.2 ± 1.0 cm) possibly as a result of a smaller heart size in women.

### Conventional coronary angiography

Conventional angiography was performed using standard techniques (Integris 3000, Philips Medical Systems, Best, the Netherlands) with the transfemoral approach after intracoronary administration of 0.1 to 0.15 mg nitroglycerin within 14 days after MSCT. Quantitative coronary angiography was done by using two orthogonal projections to identify significant diameter reductions (at least 50%) in all 15 coronary artery segments [15]. The diameter of the reference vessel on conventional coronary angiography had to measure at least 1.5 mm for a stenosis to be included in the analysis of diagnostic accuracy of MSCT, thus covering all stenoses that are possible targets for revascularization. If a coronary artery contained more than one significant stenosis, the most proximal one determined the diagnostic accuracy for the assessment of that coronary artery, since restricted flow resulting from a proximal stenosis can limit assessment of distal stenoses on noninvasive imaging [2].

### Data and image analysis

The results of conventional coronary angiography served as the reference standard for assessing the per-patient sensitivity, specificity, accuracy, nondiagnostic rate, and negative and positive predictive values of CT for detection of significant coronary stenoses (at least 50% diameter reduction, as described above) in each patient in an intention-to-diagnose design (all patients were included regardless of image quality) [12]. Nondiagnostic patients were defined as patients with no stenosis seen on MSCT and at least one main coronary branch with nondiagnostic image quality (relevant motion artifacts or decreased contrast precluding evaluation for the presence of stenoses in at least one segment in this main coronary branch). CT image analysis was performed using an automatic vessel detection tool with curved multiplanar reformations along the vessels and orthogonal cross-sections [16].

Effective radiation exposure during MSCT was estimated for all patients on the basis of individual helical scan lengths using CT-Expo 1.3 [17]. Using the same software, relative organ doses contributing to the effective dose were estimated for women and men with a scan range of 10 cm and the CT scanning parameters described above. Image noise, contrast-to-noise ratios, the coronary vessel lengths, and the relative vessel lengths free of motion artifacts (vessel contour uninterrupted and clearly delineated) were estimated on curved multiplanar reformations of MSCT as recently described in detail elsewhere [7,18]. Briefly, image noise was measured as the SD of density in a 10-mm^2 ^region-of-interest in the ascending aorta and contrast was calculated by dividing densities in 5-mm^2 ^regions-of-interest in the proximal vessel segments by the densities in surrounding tissue. No minimum vessel size was used for the analysis of vessel lengths. In addition, the diameters of all four main coronary branches (LMA = left main coronary artery, LAD = left anterior descending coronary artery, LCX = left circumflex coronary artery, RCA = right coronary artery) were measured on orthogonal cross-sections 5 mm from the origin using the automatically generated curved multiplanar reformations. Coronary artery diameters were compared between genders to analyze whether coronary artery size might explain gender differences in diagnostic accuracy. Coronary diameters were also compared after normalization for body surface area (in m^2^) determined using the formula of DuBoys and DuBoys [19]. Finally, vessel wall calcification was compared between genders and classified visually as either no calcification, calcium spots (small isolated eccentric lesions), moderate calcification, or severe calcification (large high-density lesions extending along the vessel wall).

### Statistical analysis

All data are expressed as means ± SD. A contingency analysis with a χ^2 ^or Fisher exact test was used to compare the diagnostic accuracy of MSCT in women and men. The unpaired t-test was used to compare the examination parameters of MSCT and conventional coronary angiography, the coronary vessel lengths, noise values, and the contrast-to-noise ratios for women vs. men. The chi-square and the unpaired t-test were also used to compare nonparametric and parametric image and patient characteristics. No post-hoc power analysis was performed. A *p *value < 0.05 was considered statistically significant.

## Results

### Coronary artery parameters

**Figure 1:**
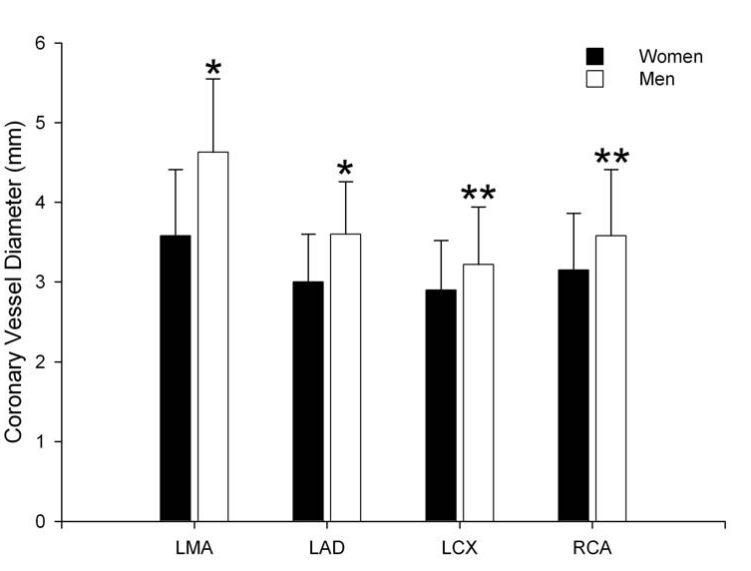
**Comparison of the coronary artery diameters in women and men**. All coronary artery diameters on MSCT were larger in men with two different levels of significance (* indicates *p *< 0.001; ** indicates *p *= 0.001). LMA indicates left main coronary artery, LAD indicates left anterior descending coronary artery, LCX indicates left circumflex coronary artery, RCA indicates right coronary artery.

The coronary artery diameters were significantly larger in men than women for all four main coronary artery branches (Fig. 1). The coronary artery diameters of men were on average larger by 20–25% for the LMA and LAD (*p *< 0.001) and 14–17% for the LCX and RCA (*p *= 0.001 and *p *< 0.001, Fig. 1, Table 2). The body surface area (1.99 ± 0.18 m^2 ^vs. 1.76 ± 0.12 m^2^, *p *< 0.001) was significantly higher in men than women. After normalization for body surface area the coronary artery diameters were not significantly different with the exception of the LMA (Table 2).

**Table 2:** Comparison of coronary artery diameters without and after normalization for body surface area (per 1 m^2^) in women and men

	Women (n = 50)	Men (n = 95)	p
Without normalization			
LMA	3.7 ± 0.7 mm	4.6 ± 0.9 mm	<0.001
LAD	3.0 ± 0.6 mm	3.6 ± 0.7 mm	<0.001
LCX	2.8 ± 0.6 mm	3.2 ± 0.7 mm	= 0.001
RCA	3.0 ± 0.6 mm	3.6 ± 0.8 mm	<0.001
After normalization for body surface area			
LMA	2.1 ± 0.4 mm/m^2^	2.3 ± 0.5 mm/m^2^	<0.01
LAD	1.7 ± 0.4 mm/m^2^	1.8 ± 0.4 mm/m^2^	0.051*
LCX	1.6 ± 0.3 mm/m^2^	1.6 ± 0.4 mm/m^2^	0.794*
RCA	1.7 ± 0.4 mm/m^2^	1.8 ± 0.4 mm/m^2^	0.323*

* not significant.

**Figure 2:**
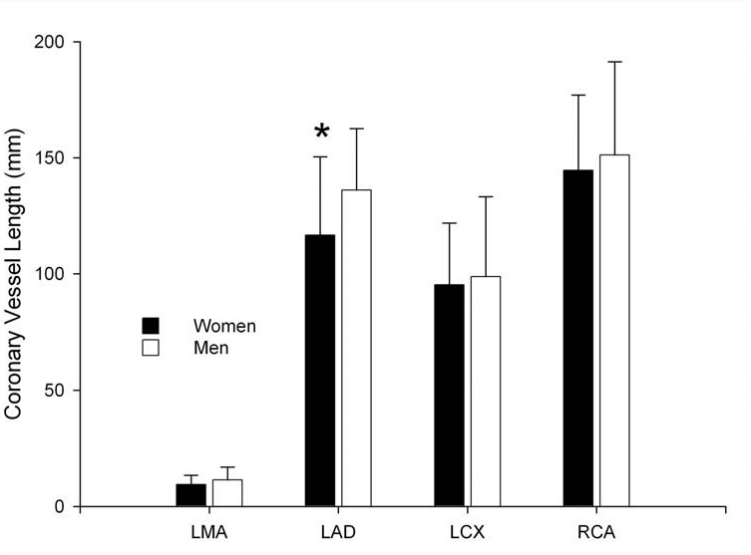
**Comparison of the entire coronary vessel lengths in women and men**. The LAD (asterisk) was significantly (*p *< 0.01) shorter in women compared with men on MSCT. LMA indicates left main coronary artery, LAD indicates left anterior descending coronary artery, LCX indicates left circumflex coronary artery, RCA indicates right coronary artery.

All four coronary vessels were shorter in women than men, with significance for the LAD (118.2 ± 35.4 mm vs. 136.1 ± 26.5 mm, *p *< 0.01, Fig. 2). Also, the vessel length free of motion artifacts tended to be shorter in women than men: LMA (10.4 ± 4.3 mm vs. 11.4 ± 5.6 mm, *p *= 0.269), LAD (114.2 ± 36.2 mm vs. 129.6 ± 33.1 mm, *p *= 0.01), LCX (85.9 ± 30.3 mm vs. 90.0 ± 38.1 mm, *p *= 0.511), and RCA (120.7 ± 52.2 mm vs. 124.7 ± 60.2 mm, *p *= 0.698). Image noise did not show a significant difference between women (18.6 ± 2.8) and men (17.8 ± 4.7, *p *= 0.523). Similarly, the contrast-to-noise ratios were not significantly different between women and men with the following values for the four coronary arteries for women and men: LMA (19.3 ± 4.2 vs. 19.3 ± 5.8), LAD (19.5 ± 5.5 vs. 19.1 ± 5.7), LCX (17.5 ± 3.5 vs. 17.6 ± 5.6), and RCA (18.3 ± 3.2 vs. 18.1 ± 5.8).

### Coronary wall calcifications

Coronary calcifications were significantly more common and more extensive in men compared with women (Table 3). On average there were small, moderate, and severe calcifications in 1.0, 0.7, and 0.3 coronary segments in women, whereas in men such calcifications could be found on average in 2.0, 1.7, and 0.7 segments per patient. Forty-four percent of the women but only 17% of the men had no coronary calcifications at all (Table 3).

**Table 3:** Comparison of coronary artery calcifications in women and men

	Women (n = 50)	Men (n = 95)	p*
Per-patient analysis^†^	50 patients	95 patients	= 0.001
No calcification	22/50 (44%)	16/95 (17%)	
Small calcified lesion	13/50 (26%)	18/95 (19%)	
Moderate calcification	8/50 (16%)	29/95 (31%)	
Severe calcification	7/50 (14%)	32/95 (34%)	
			
Per-segment analysis^$^	750 segments	1425 segments	< 0.001
No calcification	646/750 (86%)	1011/1425 (71%)	
Small calcified lesion	51/750 (7%)	188/1425 (13%)	
Moderate calcification	36/750 (5%)	162/1425 (11%)	
Severe calcification	17/750 (2%)	64/1425 (4%)	

* compared using the chi-square test.

^† ^The maximum coronary calcification seen in any of the 15 segments was used for the per-patient comparison.

^$ ^15 segments per patient.

Because of rounding percentages may not total 100%.

### Diagnostic accuracy

The accuracy of MSCT for the identification of patients with coronary artery disease as assessed by conventional coronary angiography was significantly lower for women (72%) compared with men (89%, *p *< 0.01, Table 4). Also sensitivity (70% vs. 95%), positive predictive value (64% vs. 93%), and the rate of nondiagnostic examinations (14% vs. 4%, all: *p *< 0.05, Table 4) were significantly worse for women. The reason for the significantly higher nondiagnostic rate in women were cardiac motion-related artifacts affecting the right coronary artery in five female patients. Both the false-positive and false-negative findings were significantly influenced by the presence of coronary calcium (*p *< 0.01, chi-square test) – 16 of the 19 false-positive lesions in the study cohort (84%) showed coronary calcification with 9 (47%) of them being severely calcified, whereas only 7 of the 16 false-negative lesions (44%) were calcified (none of them severely). This difference between false-positive and false-negative lesions was similar for the genders.

**Table 4:** Comparison of MSCT coronary angiography in women and men

	Women (n = 50)	Men (n = 95)	p
	no./total no. (%)		
Accuracy	36/50 (72%)	85/95 (89%)	< 0.01
Sensitivity	7/10 (70%)	56/59 (95%)	<0.05
Specificity	29/40 (72%)	29/36 (81%)	0.410*
Nondiagnostic	7/50 (14%)	4/95 (4%)	< 0.05
Negative predictive value	29/32 (90%)	29/31 (94%)	0.515*
Positive predictive value	7/11 (64%)	56/60 (93%)	< 0.05

* not significant.

### Radiation dose

**Figure 3:**
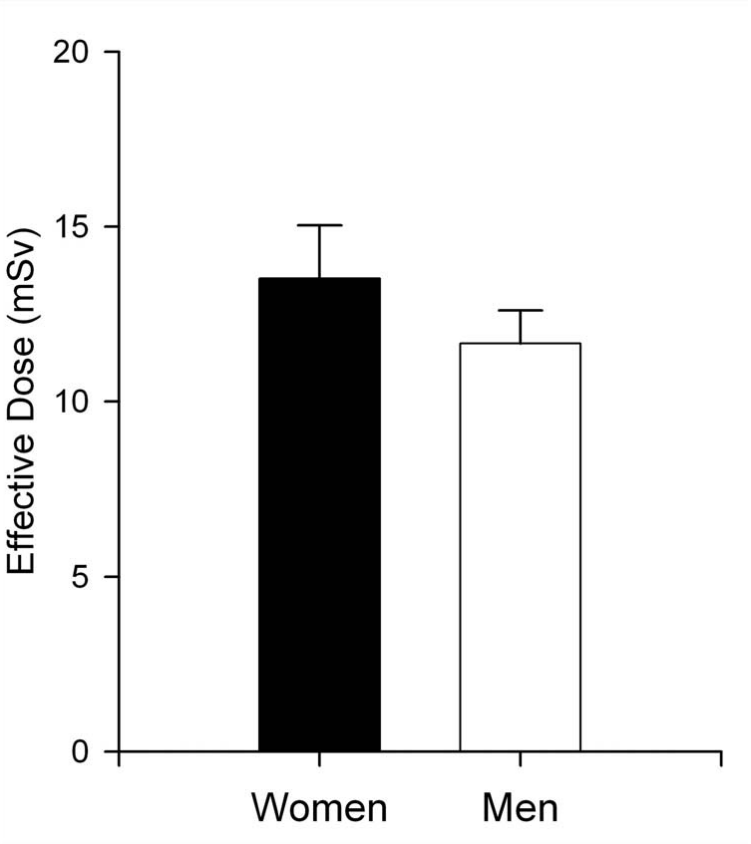
**Radiation dose comparison**. Significantly higher effective radiation dose (in mSv) of MSCT coronary angiography for women (13.7 ± 1.2 mSv) compared with men (11.7 ± 0.9 mSv, *p *< 0.001).

The effective radiation dose of MSCT coronary angiography is still one of the major issues that might limit the application of this new technology and was significantly higher by approximately 17% for the examination of women (Fig. 3). The organ dose of the breast contributed 24.5% of the effective dose in women (equal to 3.35 mSv on average) and was thus the second largest contributor to dose in women. The highest organ dose was applied to the lungs in both women (average of 5.2 mSv, 37.8% of the effective dose) and men (average of 6.2 mSv, 53.0% of the effective dose), whereas the dose to the gonads was minimal both in women (ovaries, 0.15% of the effective dose) and men (testes, < 0.1% of the effective dose). The second largest contributor to the effective dose in men was the bone marrow (0.9 mSv, 8% of the effective dose).

### Examination parameters

We observed no relevant and significant differences in other examination parameters between women and men: contrast agent amount for MSCT (107.5 ± 7.6 ml vs. 109.2 ± 11.4 ml), contrast agent amount for conventional coronary angiography (91.9 ± 13.3 ml vs. 95.8 ± 23.5 ml), room time required for MSCT (17.2 ± 3.4 min vs. 17.3 ± 6.2 min), and room time required for conventional coronary angiography (54.8 ± 11.4 min vs. 59.4 ± 17.8 min, excluding time for interventions). Also the heart rate during MSCT coronary angiography, which appears to affect CT image quality, was not significantly different between women (71.0 ± 10.0 beats/min) and men (70.0 ± 11.8 beats/min).

## Discussion

In this study on MSCT for coronary angiography, we found a significantly lower diagnostic accuracy and per-patient sensitivity while the nondiagnostic rate and the effective radiation dose of CT was higher for women compared with men.

**Figure 4:**
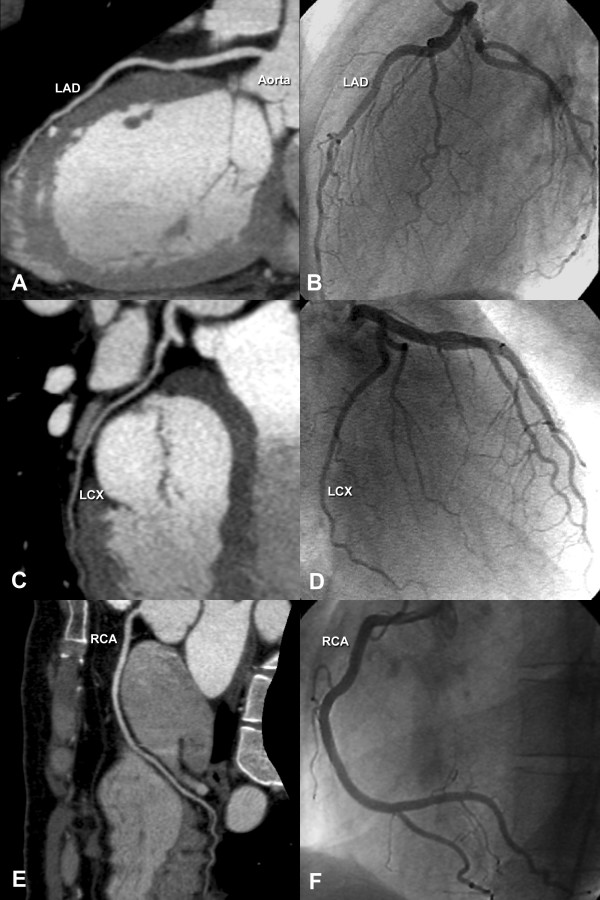
**Normal noninvasive coronary angiogram**. Obtained in a woman using MSCT with multiplanar reformations (Panels A, C, and E) in comparison to the results with conventional coronary angiography in the same patient (Panels B, D, and F). The rather small vessel sizes in women can be appreciated in this figure. LAD indicates left anterior descending coronary artery, LCX indicates left circumflex coronary artery, and RCA indicates right coronary artery.

**Figure 5:**
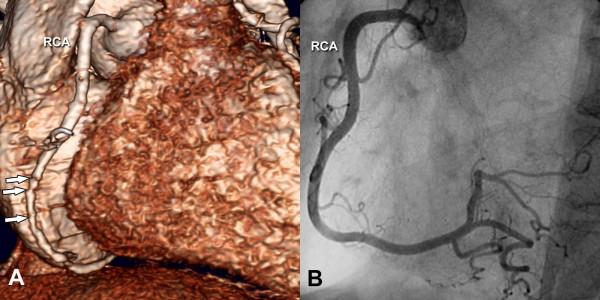
**3D reconstruction of a nondiseased RCA with motion artifacts**. Obtained in a 58-year-old woman using MSCT (Panel A) in comparison to conventional coronary angiography (Panel B). The MSCT scan shows significant stair-step motion artifacts (arrows in Panel A), which are more likely to limit diagnostic assessment if the affected vessel is small. RCA indicates right coronary artery.

From a clinical perspective the current study is of importance since there appears to be no relevant difference between the genders in the effectiveness of different therapeutic options for coronary artery disease [10]. However, there is a referral bias to conventional coronary angiography with a disadvantage to women [9]. Thus, new approaches to the diagnosis of coronary artery disease that have the potential to minimize this diagnostic gender bias are highly desirable. To reliably exclude the presence of coronary artery stenoses is the primary aim of noninvasive coronary angiography using MSCT (Fig. 4), and a number of studies have shown that because of its consistently high per-patient negative predictive value [2,4-6,11,20], MSCT might be of potential clinical value in ruling out coronary disease especially in patients with a low-to-intermediate likelihood of disease [8]. Nevertheless, the results of this study suggest that women might not benefit from this most promising candidate for noninvasive coronary angiography (MSCT) as much as men do. One possible explanation for the lower diagnostic accuracy and sensitivity in women is the significantly smaller diameter of all four coronary arteries in women than men. The smaller coronary vessel diameters in women are most likely due to body size differences. However, a lower response of women to nitroglycerin with less marked coronary dilatation cannot be excluded as another potential influencing factor. It must be remembered that the CT coronary angiography protocol used for this analysis despite the use of only 16 simultaneous detector rows enables the highest spatial resolution available [21]. The larger coronary size in men facilitates image assessment and might result in fewer relevant motion artifacts. In contrast, small coronary vessels are more susceptible to "stair-step" motion artifacts, which were the foremost reason for a significantly higher nondiagnostic rate among women (Fig. 5). This is especially important because the slice thickness of MSCT (0.5 to 0.75 mm) is still considerably larger than the spatial resolution of conventional coronary angiography (0.1 mm). Our study is in contrast to some other studies showing that even the weight-adapted coronary artery size is smaller in women than men [22,23], while others have found no difference in coronary size between genders at all [24]. We measured the coronary diameter on orthogonal cross-sections and after normalization for body surface area no significant difference (except the LMA) was seen between the genders indicating that body size differences and not reduced responsiveness of women to nitroglycerin are likely the reason for the gender difference in coronary diameters. This difference was also found when only the 76 patients without significant coronary disease were compared (data not shown).

In terms of image quality, it is important to compare the present image parameter results with those obtained in other studies to exclude that a potential technical insufficiency caused the differences between genders. Both the contrast-to-noise ratios and the coronary vessel lengths visualized without artifacts were similar [7,25] or higher compared to those reported elsewhere [26]. Moreover, no difference between the genders was observed in regards to image noise and contrast-to-noise ratios and thus, an influence is unlikely. Coronary calcium is often assumed to preclude a high diagnostic accuracy of MSCT coronary angiography, but the only comparative study thus far has found no relevant difference in diagnostic accuracy between patients with and without relevant coronary calcifications [27]. Also calcium appears to not reduce the ability of MSCT to quanitify significant coronary stenoses [21]. In the present study calcium was not equally distributed between the genders possible due to the difference in prevalence of disease. Despite this and in agreement with the analyses by Cademartiri et al. [27,28] we did not find the detection of coronary artery stenoses to be significantly impaired by the presence of extensive calcifications (most of the false-negative lesions were not calcified), whereas relevant coronary calcifications were responsible for the majority of the false-positive cases in both genders.

Previous smaller studies on MSCT coronary angiography have shown no significant differences between both genders, probably because of the smaller sample sizes [20,29]. To the best of our knowledge, the present study shows for the first time a higher diagnostic accuracy and sensitivity of MSCT for men than for women. The study is in contrast to a different analysis of 26 women that accuracy of CT coronary angiography was not significantly different from that of men [30]. Our study is also in contrast to a recent analysis of 50 women and 50 men using 64-slice CT coronary angiography [31]. This might be due to the improvements feasible with 64-slice CT coronary angiography [32,33] but could also be influenced by the fact that nondiagnostic coronary segments (due to motion artifacts) which were more prevalent in women were excluded from analysis in this study [31] (in contrast to the intention-to-diagnose principle [12] used in our study).

A lower accuracy of exercise electrocardiography in women [34] compared with men [35] has already been reported in meta-analyses. Moreover, a recent comprehensive analysis of the literature suggests that women at risk or with suspected coronary artery disease are less often referred for the appropriate diagnostic test than are men [36]. For stress testing with echocardiography and nuclear imaging, however, the evidence suggests that women, just as men, are accurately diagnosed and risk-stratified [36]. For the symptomatic women, noninvasive stress testing is generally recommended for those at intermediate risk of coronary artery disease and stress echocardiography has similar diagnostic accuracy in women and men [36]. However, there is an important limitation of stress nuclear imaging in the evaluation of symptomatic women – namely a higher rate of false-positive results due to breast attenuation and small left ventricular chamber sizes [37]. Both exercise echocardiography and nuclear imaging are limited by advanced age in women with suspected disease because exercise capacity decreases with age. Thus, pharmacologic stress testing may overcome these disadvantages in many women with suspected coronary artery disease [36]. Nevertheless, for the reasons mentioned above, testing for the presence of coronary artery disease without stress might be beneficial in women.

Two major tests have been suggested for this purpose – identification of coronary calcium on unenhanced and of coronary stenoses on contrast-enhanced CT scans. Raggi et al. recently concluded that asymptomatic women might actually benefit to a greater extent than asymptomatic men from coronary calcium scoring using computed tomography in addition to risk factor screening [38]. However, the general clinical value of calcium scoring for both genders is still under dispute especially since large randomized studies analyzing the clinical value in management are still missing [39]. For direct visualization of coronary stenoses, noninvasive coronary angiography using multislice CT is a potentially valuable strategy in patients with suspected coronary artery disease [2-7]. Our results, however, suggest that women might not benefit at present as much from noninvasive coronary angiography with MSCT as men do.

Despite the smaller scan range in women (smaller heart size) and a consequently shorter scan time the effective radiation dose was significantly larger in women than men, mainly as a result of the fact that the radiosensitive female breast (contributing to approximately one quarter of the dose in women) is in the x-ray path, further limiting the application of MSCT coronary angiography to female patients. In contrast to that, radiation exposure during conventional invasive angiography was not different between genders and was in the same ranges as reported previously [40]. The higher effective dose of coronary CT angiography in women of 13.7 ± 1.2 mSv (equal to the effective dose of 100 to 150 chest radiographs or 50 to 75 bilateral mammographies) is a cause of concern because, like younger patients, females have an increased long-term cancer risk from radiation exposure [41]. Thus the radiation risk of MSCT coronary angiography needs to be weighed against its potential clinical benefits especially in younger and female patients. However, to achieve a balanced appraisal of the radiation risks and health benefits especially in comparison to conventional coronary angiography, one must perform a net-utility analysis of life expectancy that also takes into account the short-term advantages (e.g. avoidance of the 0.11% mortality risk of invasive angiography) and clinical utility of CT coronary angiography.

Very recently 320-slice technology (single-rotation whole-heart imaging) based on acquisition of a cylindrical volume covering the entire heart has become available [42]. This technology avoids oversampling and overranging, which cause the high radiation dose of 16- and 64-CT coronary angiography [32], and thereby reduces the effective dose by at least 50% as very recently demonstrated [43] using 256 simultaneous detector rows. Moreover, cylindrical slice CT coronary angiography using a wide-area detector (with up to 320 detector rows) also has the potential to add the fourth dimension to cardiac imaging making reliable myocardial perfusion assessment a reality. In addition to radiation exposure, clinical utility, and reduced risks also cost-effectiveness of new tests such as coronary CT angiography [44] and potential to triage patients [45] needs to be included in the societal discussion about the usefulness and utility of this ascending imaging test.

### Limitations of the study

The present study is limited by its single-center design and the small number of patients (especially women) included. The prevalence and intensity (number of vessels stenosed) of coronary disease considerably varied between the genders in our study. This might have influenced the comparison of diagnostic performance. Since there is a referral bias to conventional coronary angiography with a disadvantage to women [9] and only patients who were referred to catheterization could be included in the present study such a bias might have also influenced our results. Upcoming multicenter studies of MSCT coronary angiography, such as the CorE64 trial, have the potential to further analyze the importance of the gender difference in noninvasive coronary angiography.

The first multicenter study on noninvasive coronary angiography with MSCT published thus far [46] has shown that 16-slice technology when used in several centers with varying experience is limited by a high number of uninterpretable cases and a high false-positive rate. 64-slice CT became available recently [47-52] and holds promise to increase image quality by reducing imaging time and artifacts. There is evidence from a small intraindividual study that 64-slice CT results in higher image quality for noninvasive coronary angiography than CT using 16 detector rows [32] and future studies will have to determine the value of 64-slice scanners in women.

Also dual-source CT has been shown to be a promising candidate to further reduce the length of the image reconstruction interval [53,54] and thereby improve temporal resolution and might improve the results of noninvasive coronary angiography for both men and women. No beta blockers were given prior to MSCT, which might be considered a limitation since CT coronary angiography benefits from slower heart rates [55,56]. Nevertheless, heart rate and temporal resolution were not different between genders in our study and thus are unlikely to have influenced the gender comparison of CT coronary angiography. Further improvements might be achieved in the near future using volumetric cylindrical CT coronary imaging with 256 [57] or even 320 [42] simultaneous detector rows. The present study limited the analysis to stenoses in segments with a reference vessel diameter of at least 1.5 mm. However, since smaller vessels are not easily amenable to coronary revascularization, all stenoses that might be targets for revascularization were included. The female patient cohort (19) that was added to our initial patient group [11] to increase the number of women available for comparison and thus improve scientific validity might be seen as a potential confounding factor. However, neither accuracy nor nondiagnostic rate in the initial cohort (74 and 16%) was relevantly different from the cohort of 50 women.

## Conclusion

The results of the present study show that MSCT has a lower diagnostic accuracy and sensitivity for the detection of coronary artery disease while the nondiagnostic rate is increased in women than in men compared with conventional coronary angiography as the reference standard. Also, radiation exposure from this examination is relevantly higher in women. Thus, the potential clinical benefits of MSCT coronary angiography might not be as high for the management of women with suspected coronary artery disease as for men.

## Competing interests

MD and BH have received grant support from GE Healthcare Biosciences (formerly: Amersham Buchler) for this study. The funding source had no role in the collection, analysis, or interpretation of the data or in the decision to submit the manuscript for publication. MD reports being one of the principal investigators of the CorE64 study – a multicenter trial on MSCT coronary angiography sponsored by Toshiba Medical Systems. MD receives financial support from Bracco-Altana for studying CT coronary angiography and serves as a speaker for Toshiba Medical Systems and Schering (Berlex). MD also offers hands-on cardiac CT workshops [58].

## Authors' contributions

MD conceived the idea for the study and drafted the manuscript. All authors performed data analysis and interpretation. BH provided senior guidance in all aspects. All authors reviewed and commented on the manuscript and approved the final version.

## Pre-publication history

The pre-publication history for this paper can be accessed here:


